# Interfering heterophile antibodies as the cause of persistently falsely elevated high-sensitivity troponin I on Alinity i: a case report

**DOI:** 10.11613/BM.2025.021001

**Published:** 2025-06-15

**Authors:** Ivana Lapić, Dunja Rogić, Dragana Šegulja, Sandra Jakšić Jurinjak, Željka Vogrinc, Sanja Kačkov, Fran Smaić, Indira Imširović, Lovorka Đerek

**Affiliations:** 1 Department of Laboratory Diagnostics,University Hospital Centre Zagreb, Zagreb, Croatia; 2 Faculty of Pharmacy and Biochemistry, University of Zagreb, Zagreb, Croatia; 3 Department of Cardiovascular Diseases, University Hospital Centre Zagreb, Zagreb, Croatia; 4 Medical-biochemistry Laboratory, Special Hospital Agram, Polyclinic Zagreb, Zagreb, Croatia; 5 Clinical Department for Laboratory Diagnostics, University Hospital Dubrava, Zagreb, Croatia

**Keywords:** cardiology, troponin, immunoassay, heterophile antibodies, myocarditis

## Abstract

Hereby we describe a case of a 59-year-old female patient with persistently elevated high-sensitivity troponin I (hs-TnI) over the course of almost four years measured on Alinity i with the corresponding assay (Abbott Laboratories, Chicago, USA). The patient underwent multiple extensive cardiological evaluations, but none of them suggested acute or chronic cardiac damage. Therefore, interference in measurement was suspected by the attending cardiologist and a detailed, stepwise laboratory investigation was undertaken in the sample with initial hs-TnI result of 2077 ng/L. Serial sample dilutions (1:2,1:5,1:10) did not match the expected, calculated hs-TnI concentrations, yielding both huge positive biases (62, 109 and 139%, respectively) and absolute differences (639, 453 and 290 ng/L, respectively). Precipitation with polyethylene-glycol, pretreatment in heterophilic blocking tubes (HBT) and immunoglobulin G depletion yielded hs-TnI results below the assay’s diagnostic cut-off (< 15.6 ng/L). Alternate hs-TnI immunoassays (Siemens Healthineers, Beckman Coulter and Snibe) and measurement with the high-sensitivity troponin T (hs-TnT) assay yielded results below assays’ specific cut-off values. This investigation confirmed that results of hs-TnI obtained by the Abbott assay were spuriously elevated. Significant lowering of hs-TnI after HBT pretreatment indicated that heterophile antibodies are the most probable source of interference. Based on this finding, it was entered in the patient’s medical record that future determinations of cardiac troponin should be performed with an alternate hs-TnI or hs-TnT assay. This case emphasizes that analytical interferences are usually immunoassay-dependent. Evaluation of laboratory results in the clinical context and close collaboration between laboratory and clinical staff is crucial for their recognition.

## Graphical abstract

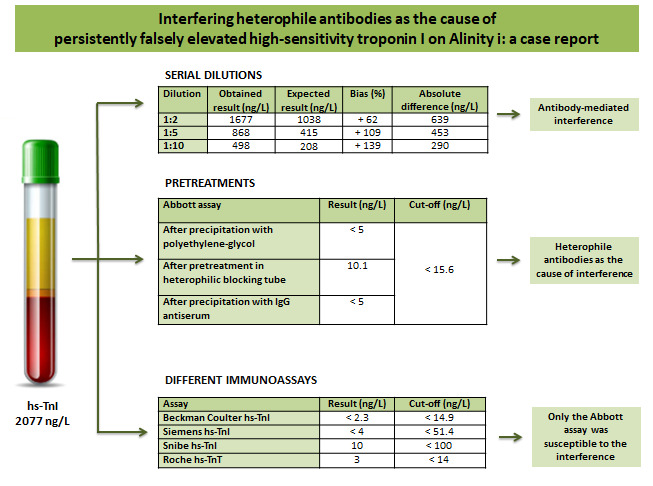

## Highlights

• Stable cardiac troponin (cTn) elevations were caused by heterophile antibodies

• Interference was limited to the Abbott high-sensitivity troponin I (hs-TnI) assay

• Interference was confirmed through detailed and stepwise laboratory investigation

• Confirmed interference was noted in patient’s medical record

• Active communication and collaboration between laboratory professionals and clinicians is crucial

## Introduction

Laboratory measurement of cardiac troponins (cTn) is pivotal in detecting myocardial injury and serial measurements are required to assess the dynamics that is associated with acute myocardial infarction (AMI) (*1*). In patients with clinical evidence of acute myocardial ischemia, a rise and/or fall of cTn with at least one result above the 99th percentile of the upper reference limit is indicative of AMI. For this purpose, both troponin I (TnI) and troponin T (TnT) measured by high-sensitivity immunoassays are equally relevant (*2*). Persistently elevated cTn values with an unchanging pattern are a reflection of conditions characterized by chronic myocardial injury, the most common being congestive heart failure, myocarditis, pulmonary embolism and end-stage kidney disease (*3*). Despite advancements in the field and remarkable performances of contemporary high-sensitivity immunoassays, falsely elevated cTn can still occur due to the presence of interfering factors in the sample (*4*, *5*). Given the urgent need for timely diagnosis of AMI, any patient with elevated cTn is promptly evaluated. In cases when this elevation is a consequence of an analytical interference rather than an underlying pathophysiological condition, the patient might undergo unnecessary diagnostic procedures and medical treatment, which compromizes patient safety, causes discomfort and can even lead to life-threatening complications (*5*). Therefore, in cases of a suspected spurious cTn result, investigative steps for assessment of the possible interference in cTn measurement should be undertaken. In order to facilitate this process, the International Federation of Clinical Chemistry Committee on Clinical Applications of Cardiac Biomarkers (IFCC C-CB) recently published recommendations for identification of interferences affecting cTn assays (*6*).

Hereby we present a case of a patient who underwent multiple extensive cardiologic evaluations due to persistently falsely elevated high-sensitivity troponin I (hs-TnI). The protocol suggested by the IFCC C-CB was applied for recognition and clarification of the possible underlying analytical intereference.

## Case report

### Patient history

A 59-year-old female patient was admitted to the Department of Cardiovascular Diseases, University Hospital Centre Zagreb, Croatia in October 2024 for re-evaluation of her cardiology status due to persistently elevated hs-TnI concentrations without dynamic changes for the last four years. In December 2020, a month after she was affected by coronavirus 2019 (COVID-19), the patient presented to the Emergency Department with cough, fatigue and shortness of breath. Electrocardiogram was unremarkable while laboratory evaluation revealed markedly elevated hs-TnI concentrations of 3362 ng/L (assay’s specific diagnostic cut-off for females < 15.6 ng/L) without a significant change in dynamics after 3 hours, with hs-TnI being 3135 ng/L. Measurement of TnI was performed using the STAT High Sensitive Troponin-I assay by Abbott Laboratories, Chicago, USA on the dedicated Alinity i analyzer (Abbott Laboratories, Chicago, USA) (*7*). The assay relies on the chemiluminesce microparticle immunoassay (CMIA) principle. Laboratory evaluation included D-dimers, measured by the automated immunoturbidimetric assay INNOVANCE D-dimer (Siemens Healthineers, Marburg, Germany) on the automated coagulation analyzer Sysmex CS-5100 (Siemens Healthineers, Marburg, Germany), which were slightly increased (0.67 mg/L FEU, cut-off < 0.50 mg/L FEU). Results of all other performed laboratory tests including complete blood count, emergency clinical chemistry tests, prothrombin time, activated partial thromboplastin time and dipstick urinalysis were within reference intervals. The working diagnosis which had been based solely on hs-TnI result was post-COVID-19 myocarditis. The patient was admitted and an elaborate cardiological evaluation followed.

Coronary angiography excluded stenosis of coronary arteries, cardiac magnetic resonance imaging (MRI) revealed a structurally healthy heart with normal anatomy and function, without any signs of myocarditis, while pulmonary emobolism was excluded by normal findings of computed tomography pulmonary angiography (CTPA). Transthoracic echocardiogram also did not reveal any structural abnormalities of the heart, however a minor pericardial effusion without hemodynamic repercussions was observed. Arterial hypertension was confirmed at that time. During hospitalization, hs-TnI was repeated and the obtained result was 3593 ng/L. In addition, N-terminal prohormone of brain natriuretic peptide (NT-proBNP) was 368 ng/L at hospital admission and 74 ng/L four days later.

Based on the results of the diagnostic evaluation, it was assumed that elevated hs-TnI concentrations are a consequence of a transient myocardial necrosis due to COVID-19-induced myocarditis and the patient was discharged. The following medications were introduced: bisoprolol (2.5 mg), pantoprazole (20 mg 1x1), acetylsalicylic acid (100 mg 1x1) and a pressurised inhalation solution that per actuation contains 100 mg of beclometasone dipropionate and 6 mg of formoterol fumarate dihydrate.

### Patient monitoring

Almost for four full years after the initial cardiology workup, the patient’s health status was regularly monitored at the ambulatory clinic and at the Department of Cardiovascular Disorders, University Hospital Centre Zagreb, Croatia. During that period, the patient continuously experienced fatigue and shortness of breath and thus underwent a series of repeated diagnostic procedures: ergometric test, heart MRI, heart ultrasound and CTPA. The results of her diagnostic evaluation persistenly revealed hypertensive heart disease with minor pericardial effusion, but without acute cardiac events.

During that period, hs-TnI concentrations were measured several times using the Abbott hs-TnI assay and each measurement revealed values largely exceeding the assay’s diagnostic cut-off. Occasionally, determinations of CK and NT-proBNP were also requested, and NT-proBNP concentrations were slightly above the cut-off, which was in conjuction with the evidenced hypertension and minor pericardial effusion. The longitudinal results of hs-TnI by the Abbott assay, CK and NT-proBNP are presented in Table 1.

**Table 1:** Longitudinal results of high sensitivity troponin I measured by the Abbott assay, creatine kinase and N-terminal prohormone of brain natriuretic peptide

**Parameter** **(unit)**	**Reference** **interval**	**24/03/2021**	**25/06/2021**	**11/08/2021**	**03/01/2022**	**06/03/2023**	**29/05/2024**	**16/10/2024**
hs-TnI (ng/L)	< 15.6	2886	2588	2928	3358	3217	2472	2077
CK (U/L)	< 153	N/A	N/A	N/A	N/A	131	125	154
NT-proBNP (ng/L)	< 125.0	83	N/A	206	136	N/A	180	218
hs-TnI - high-sensitivity troponin I. CK - creatine kinase. NT-proBNP - N-terminal prohormone of brain natriuretic peptide. N/A - not available.								

Additional laboratory evaluation revealed normal serum protein electrophoresis by means of capillary electrophoresis, results of immunoglobulin G, M and A measured immunoturbidimetrically were within reference ranges and antinuclear antibodies assessed by indirect immunofluoresce assay were negative.

After the last grossly elevated hs-TnI result in October 2024, the attending cardiologist contacted the laboratory and expressed doubts about the reliability of the reported hs-TnI results over time, suspecting an interference in measurement.

### Laboratory investigations

For investigation of the possible presence of interference in the sample, the recommended approach by the International Federation of Clinical Chemistry Committee on Clinical Applications of Cardiac Biomarkers was followed as much as possible (*6*). All analyses were performed from a single 3 mL VACUETTE tube containing lithium heparin as the anticoagulant (Greiner Bio-One, Kremsmünster, Austria), and plasma was obtained by centrifugation at 2100xg for 10 minutes.

Firstly, three dilutions (1:2, 1:5 and 1:10) of the native sample with hsTnI result of 2077 ng/L were prepared with manufacturer’s original diluent and re-analyzed with the Abbott hs-TnI assay. The obtained results did not match the expected, calculated hs-TnI concentration in these dilutions, with a huge positive bias and huge absolute differences from the expected values obtained for all three dilutions (Table 2). Bias was calculated according to the following formula: ((obtained result - expected result) / expected result) x 100.

**Table 2:** Results of serial dilutions of high-sensitivity troponin I measured by the Abbott assay

**Dilution**	**Obtained result (ng/L)**	**Expected result (ng/L)**	**Bias (%)**	**Absolute difference (ng/L)**
Native	2077	N/A	N/A	N/A
1:2	1677	1038	+ 62	639
1:5	868	415	+ 109	453
1:10	498	208	+ 139	290
N/A - not applicable.				

Further protocol included determination of hs-TnI with the Abbott assay after precipitation with polyethylene-glycol (PEG), pretreatment in heterophilic blocking tubes (HBT) and immunoglobulin G (IgG) depletion. Precipitation with PEG was done by mixing 200 μL of patient’s plasma and 200 μL of 25% PEG solution prepared from the stock solution PEG 6000 (Sigma-Aldrich, St Louis, USA), vortexing of the mixture, incubation for 10 minutes at room temperature and centrifugation for 10 minutes at 9184xg. Analysis was performed from the supernatant. Pretreatment of the sample using HBT (Scantibodies Laboratory, Santee, California, USA) was done following the original manufacturer’s protocol (*8*). Specifically, 500 μl of plasma sample was added to the tube, and after five complete inversions to ensure mixing with the blocking reagent at the bottom of the HBT, the sample was incubated for one hour at room temperature prior to analysis. IgG depletion was performed by mixing 200 μl of patient’s plasma sample with 20 μL of human IgG antiserum from the Antisera and Fixative kit (Sebia, Lisses, France) used for immunofixation procedure. Re-analysis of hs-TnI was done in the supernatant after 10-minute incubation. All aforementioned analyses were performed using the same hs-TnI reagent lot.

In addition, on the same day, the remaining plasma was aliquoted and one single plasma aliquot was delivered in a consecutive order to three laboratories that utilize different hs-TnI assays and one laboratory for determination of hs-TnT. The following hs-TnI assays were used: access hsTnI on the UniCel DxI 600 Access immunoanalyzer (Beckman Coulter, Brea, USA), LOCI hs-TnI assay on Dimension EXL with LM (Siemens Healthineers, Marburg, Germany), and Maglumi hs-cTnI on the Maglumi 800 immunoanalyzer (Snibe, Shenzhen, China) that are all based on the chemiluminescence immunoassay principle. For determination of hs-TnT, the Elecsys Troponin T hs on the Cobas e411 immunoanalyzer (Roche Diagnostics, Basel, Switzerland) that is based on the electrochemiluminesce immunoassay principle was used.

The results of hs-TnI measurement with the Abbott assay after applied pretreatments and results of analyses with other hs-Tn assays are presented in Table 3. A significant decrease of hs-TnI below the assay-specific 99th percentile was obtained after PEG precipitation, HBT pretreatment and IgG depletion, indicating that heterophile antibodies are the most probable cause of falsely elevated hs-TnI with the Abbott assay. Analyses of hs-TnI with assays from other manufacturers yielded results below assay-specific cut-offs, pointing out that in this particular case only the Abbott hs-TnI assay was susceptible to the interference with heterophile antibodies present in the sample. In addition, a hs-TnT value below assay’s specific cut-off was obtained. According to these findings, it was clearly stated in the patient’s laboratory report that future determinations should be performed with an alternate cTn assay.

**Table 3:** Results of cardiac troponin measurements in the native sample, after different pretreatments and with different high-sensitivity immunoassays

**Assay**	**Result (ng/L)**	**Assay-specific cut-off (ng/L)**
Abbott hs-TnI native sample	2077	< 15.6
Abbott hs-TnI after precipitation with PEG	< 5	< 15.6
Abbott hs-TnI after pretreatment in HBT	10.1	< 15.6
Abbott hs-TnI after precipitation with IgG antiserum	< 5	< 15.6
Beckman Coulter hs-TnI	< 2.3	< 14.9
Siemens hs-TnI	< 4	< 51.4
Snibe hs-TnI	10	< 100
Roche hs-TnT	3	< 14
PEG - polyethylene glycol. HBT - heterophilic blocking tube. IgG - immunoglobulin G. hs-TnI - high sensitivity troponin I. hs-TnT - high sensitivity troponin T.		

The source of heterophile antibodies in this case remains unclear, however, two possible causes arose through detailed investigation of patient’s demographic and clinical data. A month before the first hs-TnI determination the patient was affected by COVID-19, which could have induced production of heterophile antibodies (*9*, *10*). A second probable source of heterophile antibodies is her lifelong direct contact with farm and domestic animals, including hens, cows, pigs, dogs and cats.

The patient gave written informed consent for anonymous publication of medical data within this case report.

## What happened?

The presence of heterophile antibodies in patient samples interfered with measurement of hs-TnI with the Abbott assay and yielded persistent false elevations of results. Erroneous reporting of elevated hs-TnI for this patient triggered multiple, extensive cardiological evaluations as well as prolonged periods of sick leave.

## Discussion

All immunoassays are susceptible to analytical interferences that can yield erroneous results and jeopardize clinical decision making (*6*, *11*). Their effect is unpredictable and cannot be prevented by visual or optical inspection, hence representing a true analytical challenge in laboratory practice. The presence of interfering factors should be suspected anytime when a significant discrepancy between laboratory testing results and clinical findings is observed (*9*-*11*). Falsely elevated cTn results due to an unrecognized interference are especially threatening since cTn is the biochemical gold standard for diagnosing AMI. Therefore, when persistent cTn elevations in the absence of cardiac manifestations and without remarkable clinical findings are encountered, further investigative steps should be undertaken within the laboratory for detection of the possible interference in measurement. Stable cTn elevations are in the majority of cases antibody-mediated, and can be caused either by heterophile antibodies or macrotroponin complexes. Heterophile antibodies are immunoglobulins that crosslink the cTn assay capture and detection antibodies independently of cTn binding, hence generating cTn false signals and finally overestimating cTn values. On the other hand, macrotroponin is a long-lived complex formed between endogenous cTn-autoantibodies and cTn, that may also directly interact with immunoassays’ components and yield falsely increased values (*6*, *9*, *12*). As recommended by IFCC C-CB, identification of interferences affecting cTn assays should be performed in a stepwise manner. In the presented case, this protocol was followed as much as possible. Investigation started with serial dilutions of the native sample, which is an easy, fast and inexpensive screening method widely used for identification of the possible interference affecting immunoassay results (*6*). Sample serial dilutions revealed inconsistency of results compared with the expected values, displaying a huge bias and huge absolute differences, hence raising suspicion on an antibody-mediated interference. Precipitation with PEG, which is a simple method for removing high-molecular-weight biomolecules from the sample, caused a dramatical drop of the hs-TnI value compared to the native result, being below the assay’s limit of detection. Since PEG precipitates both macromolecular complexes and immunoglobulins, hence does not assess the origin of the interference, finding the underlying cause required further investigation (*6*, *12*). This was done by sample pretreatment in HBT, which yielded hs-TnI value below the assay’s 99th percentile. Heterophile blocking reagent within the HBT is not formulated to react with macrotroponin, therefore such lowering of hs-TnI result could not be associated with macrotroponin presence and this was excluded as the cause of interference (*6*). This was contrary to the case reported by Sušić *et al.* who evidenced a significant drop only after precipitation with PEG, but not using HBT, and concluded that macrotroponin is the most probable cause of long-term false positive hs-TnI measured by the Abbott assay (*13*). According to the obtained findings, heterophile antibodies arose as the most probable cause of interference in our case, which was further confirmed by an equal lowering of hs-TnI value after IgG depletion. If feasible, this is a valuable assessment to conduct whenever heterophile antibodies are suspected since the majority of false positive hs-TnI reported in literature are caused by interferences of circulating IgG (*6*). Furthermore, hs-TnI analyses were performed on three alternate immunoassay platforms and uniformly yielded results below the assays’ specific cut-off values. This finding once again confirms that antibody-dependent interferences are usually immunoassay-specific and that testing with alternate cTn assays is a convenient approach for elucidation of cases when an analytical interference is suspected. Although all used hs-TnI immunoassays are based on the sandwich principle and use monoclonal antibodies from animal sources, they differ by their nature and epitope specificity, therefore yielding variable susceptibility to antibody-mediated interferences. Finally, determination of hs-TnT yielded a result below the manufacturer’s 99th percentile, confirming that acute myocardial injury is highly improbable. Measurement of cTnT, if available, might be a quick and straightforward solution in such ambiguous cases, not only because a different component of the troponin complex is detected but also due to the possibly lower susceptibility of cTnT assays to analytical interferences compared to cTnI assays (*6*, *9*).

Despite continuous improvements of reagent formulations and addition of blocking agents, none of the available immunoassays on the market is exempt from heterophile antibodies interference. According to literature data, the frequency of heterophile antibodies interference in cTn assays is as high as 3.1% (*11*, *12*). Numerous cases dealing with interferences of heterophile antibodies in cTn assays, both conventional and high-sensitivity, have been published and nearly all of them report delayed recognition of the interference, consequently causing inadequate and excessive patient diagnostic management and treatment in the meantime. It was uniformly shown that suspicion of a possible falsely elevated cTn result is raised only after several measurements (*11*, *13*-*16*). Underrecognition of cTn spurious results can be partly attributed to limited awareness about the susceptibility of cTn immunoassays to interferences. However, identification of interferences affecting cTn assays can be further complicated by confounding non-specific cardiac-related symptoms due to which the patient was tested in the first place, as evidenced also in the studied case. Given the acuity of conditions associated with elevated cTn and the overall low frequency of interferences, it is reasonable to regard any cTn elevation as a possible sign of myocardial injury and refer the patient for further diagnostic evaluation (*12*). However, stable elevations of cTn over time measured by the same assay which are not in accordance with patient’s clinical condition and results of cardiological evaluation, should prompt the clinican to suspect the reliability of the result. It is of utter importance to inform clinicians that they must alert the laboratory in order to trigger investigations into presence of an analytical interference. In this particular case, two attending cardiologists separately contacted two different laboratory specialists whose number they happened to have within their phone contacts. Both laboratory specialists were not in charge for cTn testing. This clearly shows that communication channels between clinics and laboratory within our institution are still insufficient and haphazard. However, all doubtful cases such as this one can be resolved only through immediate direct collaboration between laboratory professionals and clinicians. This case shows that laboratory needs to work on visibility and establish better communication channels with clinical partners. It is important to understand that lack or delay in communication and active collaboration can cause false diagnoses that might bring significant medical and social burden to the affected patient. Besides well-known consequences including excessive diagnostic and therapeutic management, such diagnoses might keep the patient out of work for a prolonged time and might also decrease involvement in a variety of social activities.

In conclusion, we confirmed that among the used immunoassays only the Abbott hs-TnI assay was affected by the interference of heterophile antibodies in the studied patient, similarly as reported by Liu *et al*. (*14*). Thorough laboratory investigation eventually clarified the inconsistency between cardiological evaluation and hs-TnI values over the course of almost four years. The attending cardiologist was informed and advised to always monitor the patient with either hs-TnT or hs-TnI on alternate analytical platforms. As recommended by the IFCC C-CB (*6*), this finding has been stored in the patient’s medical record to be permanently available and serve as a warning for future determinations of cTn.

## What YOU should/can do in your laboratory to prevent such errors

Any cTn elevation should be regarded as a sign of myocardial injury until proven otherwise (an outlier or a consequence of the presence of an analytical interference).

Analytical interference in cTn measurement should be suspected when an inconsistency between the obtained cTn result and patient’s clinical condition is observed.

Recurrence of cTn elevations with the same assay in the absence of cardiac manifestations should raise suspicion on an antibody-mediated interference.

A stepwise laboratory investigation should be performed if an interference in immunoassays is suspected.

Interferences are usually immunoassay-dependent and analysis with an alternate immunoassay could elucidate the interfering effect. If interference is suspected for a hs-TnI assay, hs-TnT can be measured or *vice versa*.

Significant decrease of cTn value after pretreatment with heterophilic blocking reagent confirms the presence of interfering heterophile antibodies, and excludes macrotroponin as the cause of interference.

None of the available immunoassays for cTn measurement are exempt from the interference of heterophile antobodies.

If confirmed, the finding about particular assay interference and the recommendation to use alternate cTn assays should be clearly stated in patient’s medical record to serve as a warning for future cTn determinations.

Active communication between laboratory professionals and clinicians is essential for resolution of doubtful cases with a favorable outcome, and continuous efforts should be made to establish close collaboration.

## Data Availability

The data generated and analyzed in the presented study are available from the corresponding author on request.
